# Liver resection with right hepatic vein reconstruction using the internal jugular vein: a case report

**DOI:** 10.1186/s40792-016-0258-y

**Published:** 2016-11-12

**Authors:** Tomonari Shimagaki, Tomoharu Yoshizumi, Shinji Itoh, Takashi Motomura, Akihisa Nagatsu, Noboru Harada, Norifumi Harimoto, Toru Ikegami, Yuji Soejima, Yoshihiko Maehara

**Affiliations:** Department of Surgery and Science, Kyushu University, 3-1-1 Maidashi, Higashi-ku, Fukuoka, 812-8582 Japan

**Keywords:** Liver resection, Hepatic vein reconstruction, Internal jugular vein graft

## Abstract

**Background:**

The role of hepatectomy for malignant liver tumors has expanded during the past decades, and vascular reconstruction during liver resection is sometimes necessary to achieve curative surgery.

**Case presentation:**

We report a case of liver resection in a 54-year-old male who had liver metastasis that invaded the right hepatic vein. He had undergone laparoscopic low anterior resection for rectal cancer. Six months later, liver metastasis was detected. After the reduction of the tumor by preoperative chemotherapy, liver resection with right hepatic vein reconstruction using his own internal jugular vein graft was performed. The postoperative course was uneventful, and the patient was discharged 8 days after the surgery.

**Conclusions:**

Internal jugular vein grafts are superior to other types of vascular grafts for vascular reconstruction in liver surgery.

## Background

Liver resection is the best treatment for most malignant liver tumors. However, vascular involvement of the inferior vena cava, hepatocaval confluence, portal vein, or hepatic artery has been a contraindication to hepatic resection [1]. Recently, hepatectomy with vascular reconstruction has become more common because of advances in surgical technique [2]. The resected vessels can be reconstructed using various materials, such as a synthetic artificial graft [3], cryopreserved vein [4], recanalized umbilical vein, or cryopreserved aorta. Each reconstruction method has its own advantages and disadvantages. The graft for vascular reconstruction must have good patency to avoid congestion of the remaining hepatic tissue that can lead to deteriorating liver function and refractory ascites. In the setting of living donor liver transplantation, we have used internal jugular vein (IJV) grafts and have reported the benefits, especially on their viability and flexibility, which means the quality of expansion and contraction [5].

## Case presentation

**Fig. 1:**
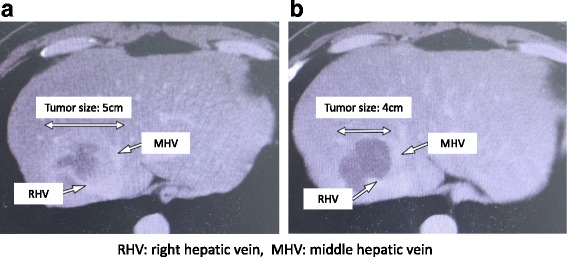
Preoperative computed tomography images of the liver, before (**a**) and after (**b**) chemotherapy. *RHV* right hepatic vein, *MHV* middle hepatic vein

**Fig. 2:**
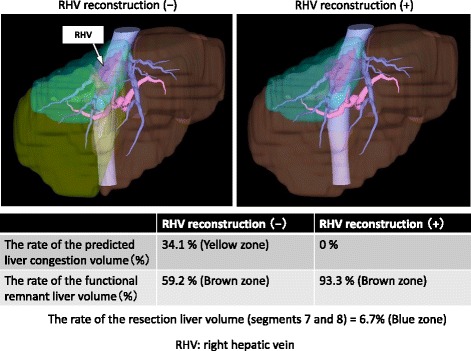
The volumetric analyses of future remnant liver; RHV reconstruction (−) and RHV reconstruction (+). The rate of the resection liver volume (segments 7 and 8) = 6.7% (blue zone). *RHV* right hepatic vein

The patient was a 54-year-old man who had undergone laparoscopic low anterior resection for rectal cancer (pT2N0M0, stage 1) 6 months previously. Liver metastasis (5 cm in diameter) was detected, with invasion of the right hepatic vein (RHV) and attachment to the middle hepatic vein. He was started on preoperative chemotherapy with mFOLFOX6 plus bevacizumab (Bmab). After seven courses of chemotherapy, the tumor size had decreased to 4 cm in diameter, and the tumor was no longer attached to the middle hepatic vein (Fig. 1). There were no complications after chemotherapy. Then, if we did not do the RHV reconstruction, the rate of the liver congestion volume would be 34.1% (Fig. 2). As a result, we decided to perform liver resection with RHV reconstruction using the IJV graft.

**Fig. 3:**
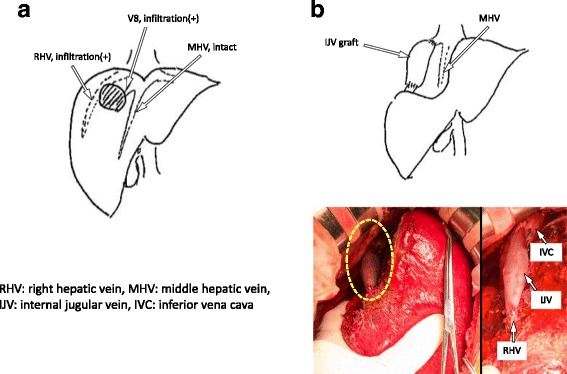
**a** Diagram showing metastatic tumor infiltration into the right hepatic vein (RHV) and V8. **b** Intraoperative image showing liver resection (segments 7 and 8) with RHV reconstruction using an internal jugular vein (IJV) graft. *MHV* middle hepatic vein, *IVC* inferior vena cava

**Fig. 4:**
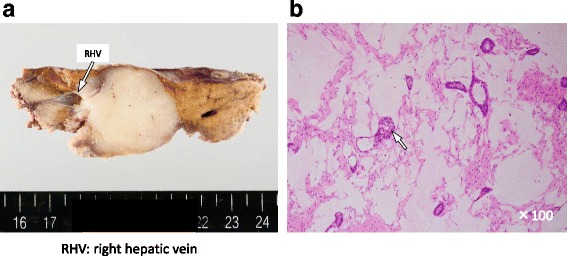
**a** Tumor, macroscopic findings. **b** Tumor, microscopic findings. Histological examination revealed moderately differentiated adenocarcinoma (*arrow*). *RHV* right hepatic vein

**Fig. 5:**
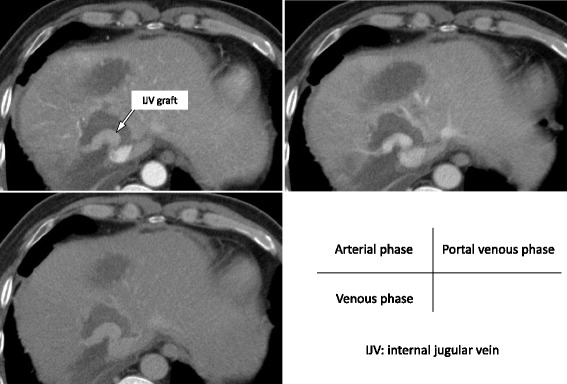
Enhanced computed tomography image of the liver in three phases on postoperative 7. The reconstructed hepatic vein was patent (*arrow*), and there was no hepatic congestion or ascites. *IJV* internal jugular vein

An approximately 6-cm-long horizontal skin incision 2 cm above the left clavicle or diagonal skin incision along the medial border of the sternocleidomastoid muscle was made. The anterior surface of the IJV was exposed by dissecting the connective tissue behind the sternocleidomastoid muscle. The omohyoid muscle was divided and the IJV was free from the surrounding tissue by dividing the small inflow branches from the level of the facial vein tributary to the level of the upper border of the clavicle. Two vascular clamps were applied on the upper and the lower portions of the dissected IJV, and the IJV graft was taken. The IJV graft was 3 cm in length and 1 cm in diameter. Then, partial hepatic resection of segments 7 and 8, including the root of the RHV, was performed. There were no tributaries of the RHV in the resected site, and the RHV was reconstructed using an IJV graft by running suture (Fig. 3). The operation time was 6 h 48 min, and the estimated blood loss was 740 ml. There was no blood transfusion during the surgery. Postoperative histopathological examination of tumor tissue revealed a moderately differentiated adenocarcinoma with invasion of the RHV (Fig. 4). The postoperative course was uneventful (Table 1). Patency of the reconstructed hepatic vein was confirmed by computed tomography on postoperative day 7. No hepatic congestion or ascites was noted (Fig. 5). He was discharged 8 days after the surgery, and he is doing well without ascites nor diuretics in the current outpatient clinic once a month. We found the graft patent by computed tomography in 3 months after the surgery.

**Table 1:** The changes of laboratory dates before and after operation

	Preoperative	POD 0	POD 1	POD 3	POD 7	POD 28
WBC (/μl)	5470	7950	7190	6170	6100	3450
T-Bil (mg/dl)	0.8	0.8	0.7	0.8	1.0	0.8
D-Bil (mg/dl)	0.1	0.4	0.2	0.1	0.1	0.2
AST (U/L)	28	1922	1972	292	45	34
ALT (U/L)	30	1599	1606	883	244	42
LDH (U/L)	264	2715	1974	326	238	225
ALP (U/L)	218	111	118	170	261	329
γ-GTP (U/L)	39	34	38	66	93	81

*POD* postoperative day

## Conclusions

Recently, the indications for surgery have evolved, shifting from oncological criteria to surgical criteria, predominantly resectability. The technical feasibility of hepatectomy has to fulfill two mandatory conditions. First, it has to achieve a completely clear macroscopic resection margin [6], and second, a sufficient volume of functioning liver has to be spared.

Vascular resection combined with liver resection is the latest step. Recently, various methods have been reported for liver resection with vascular reconstruction, such as synthetic artificial grafts, cryopreserved vein, and IJV grafts. Each graft type has its own merits and demerits. The merits of synthetic vascular grafts are their universal availability and products having various lengths and diameters, while the demerits are the relatively rigid vascular walls and tendency to become infected [7]. Infections may be refractory because of the artificial nature of the graft. Furthermore, the tendency for thrombosis of artificial grafts may require that patients take anticoagulants or antiplatelet agents [8]. The merits of cryopreserved vein are its availability in various lengths and diameters, although its use is limited to relatively few institutions. Because of the biological nature of these grafts, the tunica intima may be injured to some extent by the cold storage, freezing, and thawing processes. In addition, the homologous nature of these grafts provokes an allogeneic immune reaction, which can result in lower long-term patency rates. The most serious problem in using cryopreserved vein is the possibility of transmitting serious pathogens [9, 10].

The merits of using IJV grafts are not only the best flexibility but also the most viable vessel wall among the various vascular graft types [5]. Although the theoretical disadvantage of procuring an IJV graft is cranial vascular congestion, this has not been reported, probably as a result of the presence of many drainage veins, including the opposite IJV [10]. Another disadvantage is the resultant operative scar in the neck. In order to obtain a long IJV graft, it is usually necessary to make an approximately 6-cm-long horizontal skin incision above the clavicle or a diagonal skin incision along the medial border of the sternocleidomastoid muscle. As a countermeasure, the scar can be hidden by wearing high-collared clothing or a scarf.

In selecting the graft for hepatic vein reconstruction, the patency of the reconstructing vein is important, because the obstruction of the graft can lead to severe congestion of the remaining liver tissue, deteriorating liver function, and accumulating ascites. We believe that the IJV graft is the most useful for hepatic vein reconstruction to maintain function [11].

## Abbreviations

IJV: Internal jugular vein
RHV: Right hepatic vein

## References

[1] Hemming AW, Mekeel KL, Zendejas I, Kim RD, Sicklick JK, Reed AI (2013). Resection of the liver and inferior vena cava for hepatic malignancy. J Am Coll Surg.

[2] Soejima Y, Ueda N, Fukuhara T, Yoshizumi T, Ikegami T, Yamashita Y, Sugimachi K, Taketomi A, Maehara Y (2008). One-step venous reconstruction for a right lobe graft with multiple venous orifices in living donor liver transplantation. Liver Transpl.

[3] Jeng LB, Thorat A, Li PC, Li ML, Yang HR, Yeh CC, Chen TH, Hsu CH, Hsu SC, Poon KS (2015). “V-Plasty” technique using dual synthetic vascular grafts to reconstruct outflow channel in living donor liver transplantation. Surgery.

[4] Sugawara Y, Makuuchi M, Akamatsu N, Kishi Y, Niiya T, Kaneko J, Imamura H, Kokudo N (2004). Refinement of venous reconstruction using cryopreserved veins in right liver grafts. Liver Transpl.

[5] Uchiyama H, Shirabe K, Yoshizumi T, Ikegami T, Soejima Y, Taketomi A, Kayashima H, Morita K, Maehara Y (2012). Use of an internal jugular vein graft for middle hepatic vein tributary reconstruction in right-lobe living-donor liver transplantation. Transplantation.

[6] Gregoire E, Hoti E, Gorden DL, de la Serna S, Pascal G, Azoulay D (2010). Utility or futility of prognostic scoring systems for colorectal liver metastases in an era of advanced multimodal therapy. Eur J Surg Oncol.

[7] Jernigan TW, Croce MA, Cagiannos C, Shell DH, Handorf CR, Fabian TC (2004). Small intestinal submucosa for vascular reconstruction in the presence of gastrointestinal contamination. Ann Surg.

[8] Hwang S, Jung DH, Ha TY, Ahn CS, Moon DB, Kim KH (2012). Usability of ringed polytetrafluoroethylene grafts for middle hepatic vein reconstruction during living donor liver transplantation. Liver Transpl.

[9] Srinivasan A, Burton EC, Kuehnert MJ, Rupprecht C, Sutker WL, Ksiazek TG, Paddock CD, Guarner J, Shieh WJ, Goldsmith C, Hanlon CA, Zoretic J, Fischbach B, Niezgoda M, El-Feky WH, Orciari L, Sanchez EQ, Likos A, Klintmalm GB, Cardo D, LeDuc J, Chamberland ME, Jernigan DB, Zaki SR (2005). Rabies in Transplant Recipients Investigation Team. Transmission of rabies virus from an organ donor to four transplant recipients. N Engl J Med.

[10] Chalian AA, Anderson TD, Weinstein GS, Weber RS (2001). Internal jugular vein versus external jugular vein anastamosis: implications for successful free tissue transfer. Head Neck.

[11] Uchiyama H, Yoshizumi T, Ikegami T, Harimoto N, Itoh S, Okabe H, Soejima Y, Maehara Y. Use of internal jugular vein grafts in reconstructing multiple venous orifices of right hepatic grafts without the middle hepatic vein trunk. Liver Transpl. 2016 10.1002/lt.24644 27657354

